# Health Outcomes and Cost-effectiveness of Monoclonal SARS-CoV-2 Antibodies as Pre-exposure Prophylaxis

**DOI:** 10.1001/jamanetworkopen.2023.21985

**Published:** 2023-07-06

**Authors:** Stephanie Popping, Brooke E. Nichols, Brent Appelman, Jason J. Biemond, Magda Vergouwe, Frits R. Rosendaal, Marc van der Valk, Godelieve J. de Bree, W. Joost Wiersinga, Emma Birnie

**Affiliations:** 1 Centre for Experimental and Molecular Medicine, Amsterdam University Medical Centres—Location AMC, Amsterdam, the Netherlands; 2 Department of Medical Microbiology and Infectious Diseases, Erasmus Medical Center, Rotterdam, the Netherlands; 3 Foundation for Innovative New Diagnostics, Geneva, Switzerland; 4 Department of Medical Microbiology, Amsterdam University Medical Center, University of Amsterdam, Amsterdam, the Netherlands; 5 Department of Clinical Epidemiology, Leiden University Medical Center, Leiden, the Netherlands; 6 Stichting HIV Monitoring, Amsterdam, the Netherlands; 7 Division of Infectious Diseases, Department of Medicine, Amsterdam University Medical Centers, Amsterdam Institute for Infection and Immunity, University of Amsterdam, Amsterdam, the Netherlands

## Abstract

**Importance:**

Pre-exposure prophylaxis with neutralizing SARS-CoV-2 monoclonal antibodies (mAbs PrEP) prevents infection and reduces hospitalizations and the duration thereof for COVID-19 and death among high-risk individuals. However, reduced effectiveness due to a changing SARS-CoV-2 viral landscape and high drug prices remain substantial implementation barriers.

**Objective:**

To assess the cost-effectiveness of mAbs PrEP as COVID-19 PrEP.

**Design, Setting, and Participants:**

For this economic evaluation, a decision analytic model was developed and parameterized with health care outcome and utilization data from individuals with high risk for COVID-19. The SARS-CoV-2 infection probability, mAbs PrEP effectiveness, and drug pricing were varied. All costs were collected from a third-party payer perspective. Data were analyzed from September 2021 to December 2022.

**Main Outcomes and Measures:**

Health care outcomes including new SARS-CoV-2 infections, hospitalization, and deaths. The cost per death averted and cost-effectiveness ratios using a threshold for prevention interventions of $22 000 or less per quality-adjusted life year (QALY) gained.

**Results:**

The clinical cohort consisted of 636 individuals with COVID-19 (mean [SD] age 63 [18] years; 341 [54%] male). Most individuals were at high risk for severe COVID-19, including 137 (21%) with a body mass index of 30 or higher, 60 (9.4%) with hematological malignant neoplasm, 108 (17%) post-transplantation, and 152 (23.9%) who used immunosuppressive medication before COVID-19. Within the context of a high (18%) SARS-CoV-2 infection probability and low (25%) effectiveness the model calculated a short-term reduction of 42% ward admissions, 31% intensive care unit (ICU) admissions, and 34% deaths. Cost-saving scenarios were obtained with drug prices of $275 and 75% or higher effectiveness. With a 100% effectiveness mAbs PrEP can reduce ward admissions by 70%, ICU admissions by 97%, and deaths by 92%. Drug prices, however, need to reduce to $550 for cost-effectiveness ratios less than $22 000 per QALY gained per death averted and to $2200 for ratios between $22 000 and $88 000.

**Conclusions and Relevance:**

In this study, use of mAbs PrEP for preventing SARS-CoV-2 infections was cost-saving at the beginning of an epidemic wave (high infection probability) with 75% or higher effectiveness and drug price of $275. These results are timely and relevant for decision-makers involved in mAbs PrEP implementation. When newer mAbs PrEP combinations become available, guidance on implementation should be formulated ensuring a fast rollout. Nevertheless, advocacy for mAbs PrEP use and critical discussion on drug prices are necessary to ensuring cost-effectiveness for different epidemic settings.

## Key Points

**Question:**

Could neutralizing SARS-CoV-2 monoclonal antibodies pre-exposure prophylaxis (mAbs PrEP) be cost-effective in a population at risk for severe COVID-19?

**Findings:**

In this economic evaluation with a clinical cohort of 636 patients with SARS-CoV-2, mAbs PrEP was associated with reduced ward admissions, intensive care unit admissions, and deaths, and it was cost-saving with drug prices of $275 and 75% or higher effectiveness. A reduction of drug prices to $550 is required for cost-effectiveness ratios less than $22 000 per quality-adjusted life-years gained per death averted and to $2200 for ratios between $22 000 and $88 000.

**Meaning:**

These findings suggest that mAbs PrEP preventing SARS-CoV-2 infections can be cost-saving; however, current drug prices need adjustments to obtain cost-effectiveness ratios between $22 000 and $88 000.

## Teaser

This economic evaluation assesses the health outcomes and cost-effectiveness of monoclonal SARS-CoV-2 antibodies used as pre-exposure prophylaxis.

## Introduction

Mass vaccination has reduced the number of coronavirus disease (COVID-19) cases substantially. Nevertheless, an annual seasonal peak of Severe Acute Respiratory Syndrome Coronavirus (SARS-CoV-2) infections and, subsequently hospital admissions may be expected.^1,2^ Unvaccinated individuals or those with an inadequate vaccine immune response due to coexisting conditions will remain at an increased risk for progression to severe COVID-19 and hospitalization.^3,4^

Clinical trials among high-risk individuals showed that pre-exposure prophylaxis (PrEP) with long-acting neutralizing SARS-CoV-2 monoclonal antibodies (mAbs) can effectively prevent SARS-CoV-2 infection and reduce the risk of COVID-19 associated hospitalization and death.^5,6^ Presently, tixagevimab-cilgavimab, a neutralizing monoclonal antibody combination with an extended half-life, is the only mAbs PrEP that has received Emergency Use Authorization for PrEP or early COVID-19 treatment. Although guidelines recommend mAbs PrEP for high-risk individuals, uptake of these guidelines is slow, and mAbs PrEP is unavailable in many countries.^7,8,9^ Apart from cost as a key barrier to mAbs PrEP implementation, concerns regarding effectiveness also remain.^8,10,11^ Notwithstanding the diminished activity against the Omicron variant compared with the Wuhan and Delta variant, tixagevimab-cilgavimab remain the only available mAb recommended as PrEP.^12,13,14^

The SARS-CoV-2 viral landscape is continuously changing, resulting in a delay in availability of mAbs PrEP effectiveness data. Therefore, mathematical modeling can serve as a tool to understand the influence on new infections and hospitalization of mAbs as PrEP, with uncertainties of SARS-CoV-2 and drug price taken into consideration. For this study, a decision analytic model was constructed using clinical COVID-19 health care utilization and outcome data of individuals at high-risk for severe COVID-19 to assess the short-term health outcomes and mAbs PrEP cost-effectiveness.

## Methods

### Study Design

A decision analytic model was developed to estimate short-term (90-day) health outcomes, health care utilization costs, and cost-effectiveness associated with mAbs PrEP implementation among individuals with preexisting conditions resulting in an increased risk for severe COVID-19 (eFigure 1 in Supplement 1). We conducted the study in line with the Consolidated Health Economic Evaluation Reporting Standards (CHEERS) reporting guideline guidelines.^15^ The Amsterdam UMC Research Ethics Committee reviewed the study protocol and applied the study under the non–*Wet medisch-wetenschappelijk onderzoek* [Medical Research Involving Human Subjects Act]. Included patients provided written informed consent. The TURN-COVID study is registered at ClinicalTrials.gov (NCT05195060).

### Data Sources

#### Study Population

Our model captured data from the ongoing multicenter, prospective Dutch cohort study on neutralizing mAbs and other antiviral SARS-CoV-2 agents (TURN-COVID study).^16^ The research presented was performed in 16 Dutch hospitals (4 academic and 12 large teaching hospitals). Adults with poor response to vaccination, and therefore at increased risk for severe COVID-19, were treated with SARS-CoV-2 mAbs and invited to participate in the study. As part of routine care, eligible patients with COVID-19 in the Netherlands were treated with 2000 mg casirivimab-imdevimab (REGEN-COV) or 500 mg sotrovimab between September 2021 and April 2022.^7^ This period was characterized by the dominance of the SARS-CoV-2 Delta variant (September to December 2021) followed by the Omicron BA.1 variant (January to March 2022). Baseline characteristics, vaccination status, medical history, and medication use from participating individuals were collected from Electronic Health Records (EHRs). See eAppendix in Supplement 1 for more details.

### Health Care Utilization Costs

Health care utilization costs were captured using a microcosting (bottom-up) approach from a third-party payer perspective. We included cost of inpatient days (ward, intensive care unit [ICU], and rehabilitation), paramedical care, and care facilitated by the general practitioners’ offices. A self-completed questionnaire was requested on day 90 capturing health care utilization during the 90-day period following COVID-19. These outcomes were compared with the 90-day period preceding COVID-19 collected at day 28. Additionally, health outcomes, such as hospital admission (inpatient days, ICU admission), COVID-19 outcome (recovered or death), and discharge to rehabilitation or nursing home, were extracted from EHRs. For individuals without a SARS-CoV-2 infection, mAbs PrEP provision costs were taken into consideration. We used a time-horizon capturing the costs within the 90-day period following SARS-CoV-2 infection and due to the short time-horizon no discounting was performed. For more details on health care resources and data/cost collection, see eTable 1 and eAppendix in Supplement 1.

### Statistical Analysis

#### Decision Analytic Model and Model Assumptions

A decision analytic model was constructed to estimate the short-term health outcomes and costs of people eligible for mAbs as PrEP, including the number of new COVID-19 cases, hospital admissions (ward and ICU), deaths per 100 000 persons, and the cost per death avoided. The model started with a decision node of mAbs PrEP provision, followed by chance nodes for SARS-CoV-2 infection, hospitalization (no admission, ward admission, and ICU admission), and continued by outcome (recovery or death). Mean short-term health care utilization costs were added into the different model categories (eg, ambulatory, ward, and ICU, and considered to have mild, moderate, or severe/critical COVID-19, respectively) and outcome.

Data from the TURN-COVID cohort, the Dutch National Intensive Care Evaluation registry of admissions to ICUs,^17^ Wiersinga et al,^2^ and international studies on SARS-CoV-2 mAbs studies were used as model parameters.^5,12,18,19^ With a 100% mAbs PrEP effectiveness, the model assumed reductions of 70% in SARS-CoV-2 infection probability, 85% in ward and 94% in ICU admissions, and 65% in SARS-CoV-2–related mortality.^5,12,18^ In our model, no mAbs PrEP (baseline strategy) was compared with scenarios where mAbs PrEP was provided. Model parameters, including SARS-CoV-2 infection probability, effectiveness, and drug price were varied over the different scenarios (eTable 1 in Supplement 1).

#### Cost-effectiveness Analysis

The cost-effectiveness ratios were calculated as the difference in total costs by the difference in quality-adjusted life-years (QALYs) gained by preventing deaths over the 90-day period. Sex-weighted QALYs from Wouterse et al^20^ were used, reporting the total QALYs loss per COVID-19 death taking into account the underlying health status (3.73). As our population is heterogeneous varying with age and underlying factors influencing health-related quality of life, the number of QALYs gained per death averted was ranged (±50%). Thresholds for cost-effectiveness ratios were set at less than $22 000 (Dutch cost-effectiveness threshold for prevention interventions), $22 000 to $88 000, and more than $88 000 (Dutch cost-effectiveness threshold for treatment interventions).^21^

#### One-way Sensitivity Analysis

A 1-way sensitivity analysis was performed of the cost-effectiveness per QALY gained per death averted of mAbs PrEP vs no mAbs PrEP, considering a 50% effectiveness with a $2750 drug price, and a low infection probability as the baseline comparator. The following key input parameters were independently varied: infection probability (0.01-0.2), effectiveness (10%-100%), drug price ($100-$5000), admission probability (ward and ICU), mortality, and length of hospital stay (±50%). The statistical analysis was conducted using R version 4.1 (R Project for Statistical Computing) and Microsoft Excel version 2021 (Microsoft Corporation). Data were analyzed from September 2021 to December 2022.

## Results

### Baseline Characteristics of Study Population

In total, 636 individuals with COVID-19 were included to determine clinical outcome and health care utilization costs. Most individuals were at increased risk for severe COVID-19, including 137 (21%) with a body mass index over 30 (calculated as weight in kilograms divided by height in meters squared), 60 (9%) with hematological malignant neoplasm, 108 (17%) post-transplantation (solid and stem cell), and 152 (23%) who used immunosuppressive medication before COVID-19 (Table 1). During their SARS-CoV-2 infection, 155 (24%) were ambulatory, 366 (58%) admitted to the ward, and 115 (18%) admitted to the ICU (Table 1). Oxygen therapy was provided in over 394 (61%) of individuals (143 [22%] low flow and 251 [39%] high flow).

**Table 1.** Baseline Characteristics From 636 High-risk Individuals of the TURN-COVID Cohort

Characteristic	Patients, No (%)		
	Ambulatory (n=155)	Ward (n=366)	Intensive care unit (n=115)
Sex			
Male	63 (43.2)	200 (54.8)	78 (67.8)
Female	83 (56.8)	164 (44.9)	36 (31.3)
Age, median (IQR), y	55 (45-63)	68 (54-79)	64 (55-71)
<40	23 (15.8)	33 (9.0)	10 (8.7)
40-60	71 (48.6)	91 (24.9)	33 (28.7)
>60	52 (35.6)	241 (66.0)	72 (62.6)
Body mass index, median (IQR)^a^	25.40 (22.82-29.37)	26.79 (23.65-30.31)	27.44 (24.58-31.70)
Clinical characteristics			
SARS-CoV-2 vaccination^b^	105 (85.4)	168 (46.3)	33 (28.7)
SARS-CoV-2 seronegative (antibodies negative)^c^	109 (88.6)	333 (91.7)	110 (95.7)
High-risk factors for severe COVID-19			
Obesity (body mass index ≥30)	18 (19.6)	80 (26.8)	39 (36.4)
Cardiovascular diseases	16 (13.0)	138 (38.0)	27 (23.5)
Hypertension	76 (76.0)	152 (62.0)	57 (66.3)
Diabetes	20 (16.3)	79 (21.7)	18 (15.7)
Chronic liver disease	3 (2.4)	7 (2.0)	0 (0.0)
Chronic kidney failure	24 (19.5)	77 (21.2)	17 (18.4)
Chronic lung disease	9 (7.3)	53 (14.6)	11 (9.6)
Rheumatic disease	36 (29.3)	44 (12.1)	12 (10.4)
Inflammatory bowel disease	2 (1.6)	8 (2.2)	3 (2.6)
Solid malignant neoplasm	3 (2.8)	33 (9.9)	8 (7.3)
Hematological malignant neoplasm	21 (19.4)	30 (8.8)	9 (8.2)
Solid organ transplant	36 (29.3)	50 (13.8)	12 (10.4)
Stem cell transplant	7 (5.7)	1 (0.3)	2 (1.7)
Immunodeficiency disorders^d^	7 (5.7)	11 (3.0)	0
Any immunosuppressive medication^e^	16 (10.3)	95 (26.0)	41 (35.7)
Outcomes			
Length of hospital stay, median (IQR), d	0	7 (4-12)	18 (11-29)
Any type of oxygen therapy			
Low-flow oxygen therapy	0	139 (38.0)	4 (3.4)
High-flow oxygen therapy	0	151 (41.3)	100 (87.0)
90-d mortality	0	124 (33.9)	52 (45.2)

^a^ Body mass index is calculated as weight in kilograms divided by height in meters squared.

^b^ Individuals received at least 1 SARS-CoV-2 vaccination; unknown, 23 individuals.

^c^ Unknown, 19 individuals.

^d^ Primary and secondary immunodeficiency diseases, unknown, 1 individual.

^e^ B-and-T cell inhibitors, chemotherapy, corticosteroids, and others in the 3 months before SARS-CoV-2 infection.

### Health Care Utilization Costs

Ambulatory recovered individuals used the lowest health care costs of the 90-day period following SARS-CoV-2 infection; mean (SD) $166 ($474), median (IQR) $0 ($−1101 to $2374) (eTable 2 in Supplement 1). Among 9 individuals, the short-term health care utilization costs were lower than prior COVID-19. Compared with ambulatory recovered individuals, those recovered from the ward incurred more costs, mean (SD) $6742 ($11 101), median (IQR) $4498 ($603 to $56 060). The most expensive cost component of this group was attributed to inpatient ward days (95%). Individuals admitted to the ICU were the most expensive patient category during the first 90 days following SARS-CoV-2 infection with a mean (SD) of $39 313 ($39 229), median (IQR) $27 309 ($3017 to $197 189). Similar to the ward, the majority of costs were attributed to inpatient days (ICU and ward) (96%) and additionally to inpatient rehabilitation (2%). Within the context of a high (18%) SARS-CoV-2 infection probability and low (25%) effectiveness the model calculated a short-term reduction of 42% ward admissions, 31% intensive care unit (ICU) admissions, and 34% deaths. Cost-saving scenarios are obtained with drug prices of $275 and 75% or higher effectiveness. With a 100% effectiveness mAbs PrEP can reduce ward admissions by 70%, ICU admissions by 97%, and deaths by 92%.

### Health Outcomes After SARS-CoV-2 Infection

In the baseline scenario, the model estimated 4000 to 18 000 new SARS-CoV-2 infections per 100 000 persons over a 90-day period depending on low (0.04) or high (0.18) SARS-CoV-2 infection probability among people eligible for mAbs PrEP (Table 2). A low infection probability (eg, a declining epidemic) resulted in an estimated 760 ward and 200 ICU admissions and 259 deaths per 100 000 persons. A high probability (eg, at the beginning of an epidemic wave) resulted in a projected 3420 ward and 900 ICU admissions, and 1167 deaths per 100 000 persons.

**Table 2.** Model Estimations of New SARS-CoV-2 Infections and Health Outcomes Over the Different Scenarios

Scenario	Patients, No.			
	New SARS-CoV-2 infections	Ward admissions	Intensive care unit admissions	Deaths
Baseline, no mAbs PrEP				
Low SARS-CoV-2 probability	4000	760	200	259
High SARS-CoV-2 probability	18 000	3420	900	1167
mAbs PrEP 25%				
Low SARS-CoV-2 probability	3300	437	155	177
High SARS-CoV-2 probability	14 850	1969	699	796
mAbs PrEP 50%				
Low SARS-CoV-2 probability	2600	287	99	108
High SARS-CoV-2 probability	11 700	1290	448	486
mAbs PrEP 75%				
Low SARS-CoV-2 probability	1900	139	45	56
High SARS-CoV-2 probability	8550	625	201	252
mAbs PrEP 100%				
Low SARS-CoV-2 probability	1200	25	4	22
High SARS-CoV-2 probability	5400	113	16	98

The model varies different mAbs PrEP effectiveness (25%, 50%, 75%, and 100%) combined with a low (0.04) or high (0.18) probability of SARS-CoV-2 infection per 100 000 persons.

With a low SARS-CoV-2 infection probability, mAbs PrEP resulted in an estimated 1200 to 3300 new infections per 100 000 persons over a 90-day period for 100% to 25% effectiveness, respectively. This means a 17.5% to 70.0% reduction in infections relative to baseline. A low (25%) effectiveness yielded an estimated 437 ward admissions, 155 ICU admissions, and 177 deaths per 100 000 persons. With 100% effectiveness, mAbs PrEP provision resulted in a projected 25 ward admissions, 4 ICU admissions, and 22 deaths per 100 000 persons.

Considering a high SARS-CoV-2 infection probability, mAbs PrEP resulted in an estimated 5400 to 14 850 new infections per 100 000 persons, for a 100% and 25% effectiveness, respectively. A 25% effectiveness calculated to 1969 ward admissions, 699 ICU admissions, and 796 deaths. A 100% effectiveness resulted in a projected 113 ward admissions, 16 ICU admissions, and 98 deaths. In other words, in these scenarios the preventive effect of mAbs PrEP was more substantial, associated with a high probability of infection.

### Short-term Costs Per Death Averted After SARS-CoV-2 Infection

**Figure 1.**
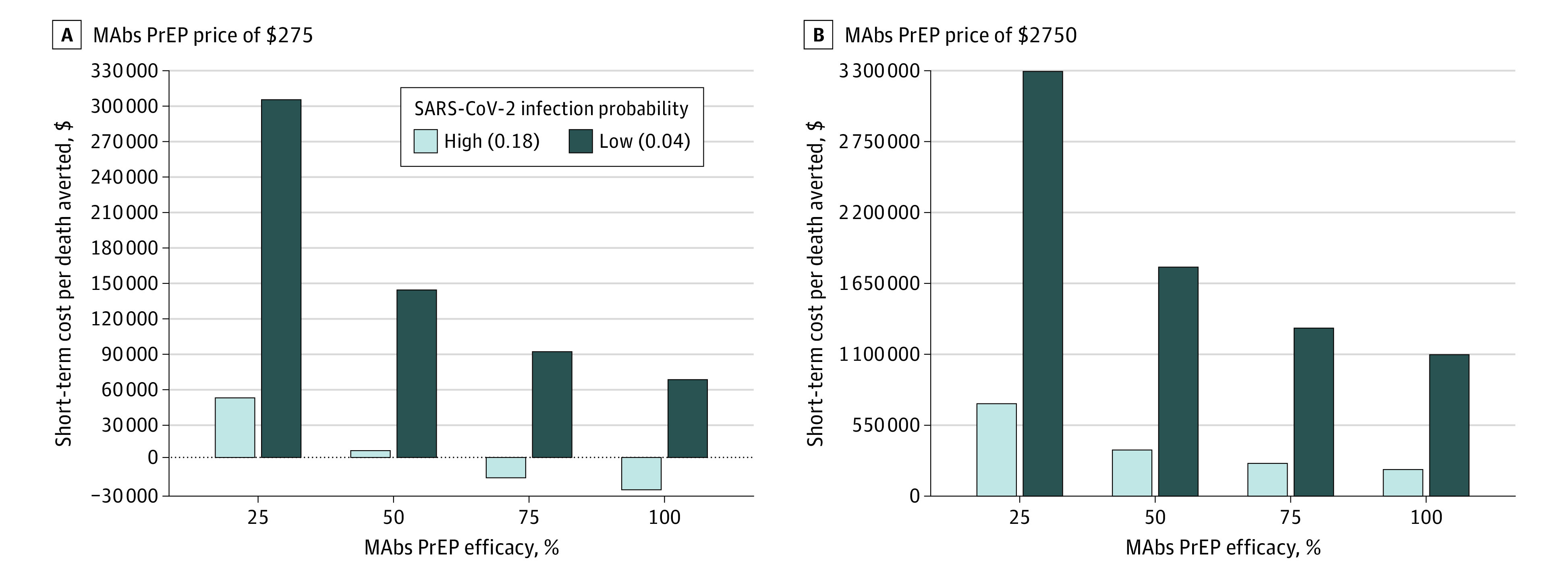
Cost Per Death Averted Varied Over Different Modeling Scenarios The y-axis shows cost per death averted in dollars, and the x-axis the monoclonal antibodies pre-exposure prophylaxis (mAbs PrEP) effectiveness.

We estimated from the model that a low ($275) mAbs PrEP price and high infection probability resulted in a cost-saving price per death averted with a minimum effectiveness of 75% (Figure 1A). However, a low infection probability increased the cost per death averted, ranging from $66 380 to $310 296 (100%-25% effectiveness).

In the scenarios with a high price ($2750), the model estimated the cost per death averted between $1 107 671 to $3 312 487 depending on the effectiveness (Figure 1B). With a high drug price, our model yielded an estimate that cost-saving scenarios per death averted are not obtained with mAbs PrEP.

### Cost-effectiveness of mAbs PrEP Among Those at High Risk For Severe COVID-19

**Figure 2.**
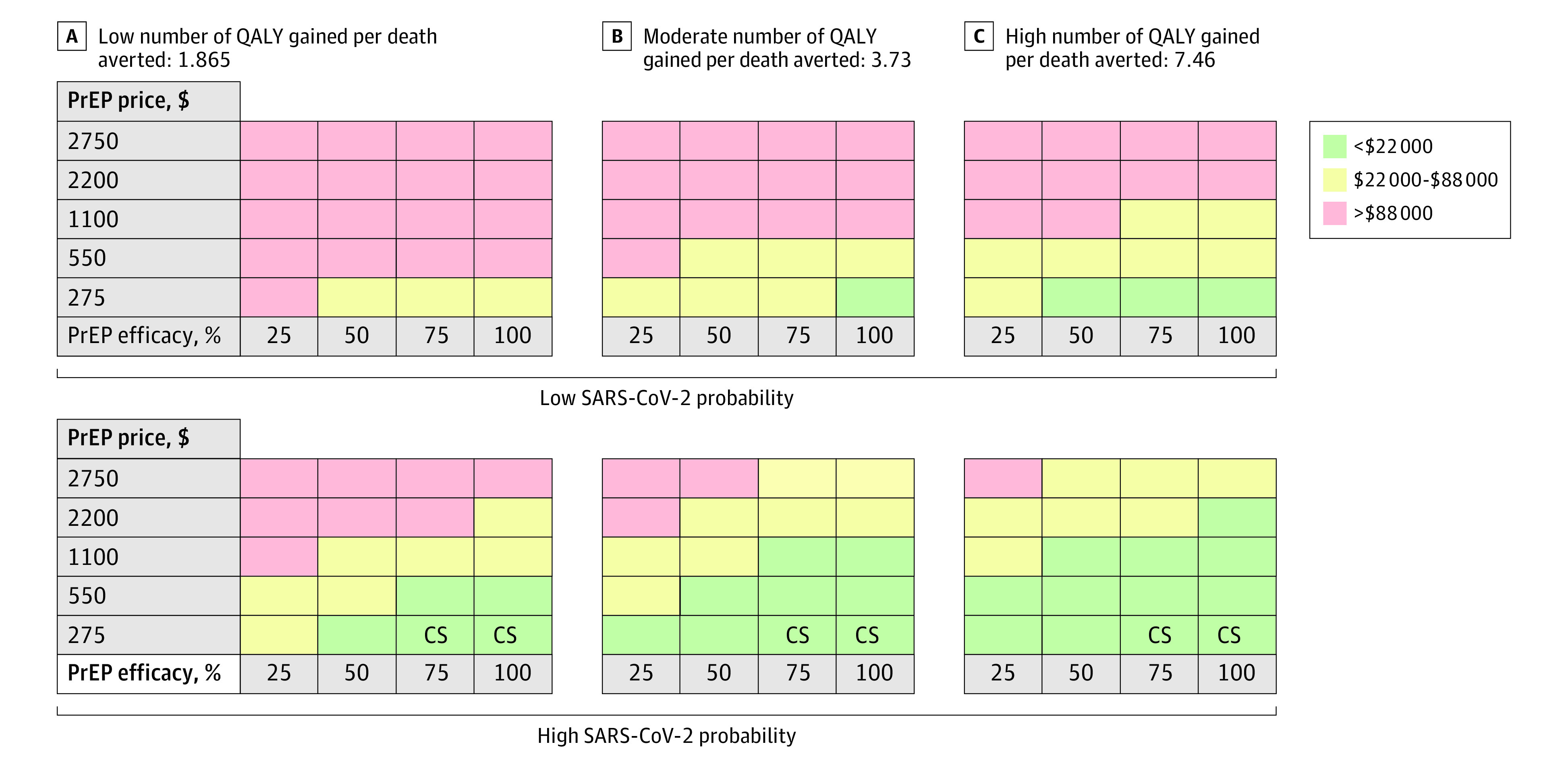
Short-term Cost-effectiveness Ratios Per Quality-Adjusted Life Year (QALY) Gained The panels in the figure shows a different number of QALY gained per death averted representing different patient populations with underlying diseases and their life expectancy. CS indicates cost-saving ($<0).^20^

According to a low infection probability, the model estimated that mAbs PrEP provision among high-risk individuals with moderate to high QALYs gained resulted in cost-effective scenarios ($<22 000) with high (50%-100%) effectiveness and a drug price of $275. Among those, ratios between $22 000 and $88 000 were obtained with drug prices between $275 and $1100. For individuals with a low number of QALYs gained (eg, elderly and those with a high number of comorbidities resulting in low quality of life and low life expectancy) a low drug price and effectiveness of 50% or less was needed for ratios between $22 000 and $88 000 (Figure 2A).

Within the context of a high SARS-CoV-2 infection probability, mAbs PrEP can be cost-saving for all individuals when drug prices are reduced to $275 and effectiveness is 75% or higher. With a low 25% effectiveness, drug prices of $550 still reach cost-effectiveness ratios between $22 000 and $88 000 for all individuals. Among individuals with moderate to high QALYs, gained ratios between $22 000 and $88 000 are obtained with drug prices from $1100 or lower, regardless of effectiveness (Figure 2B and eFigure 2 in Supplement 1).

### Sensitivity Analysis

**Figure 3.**
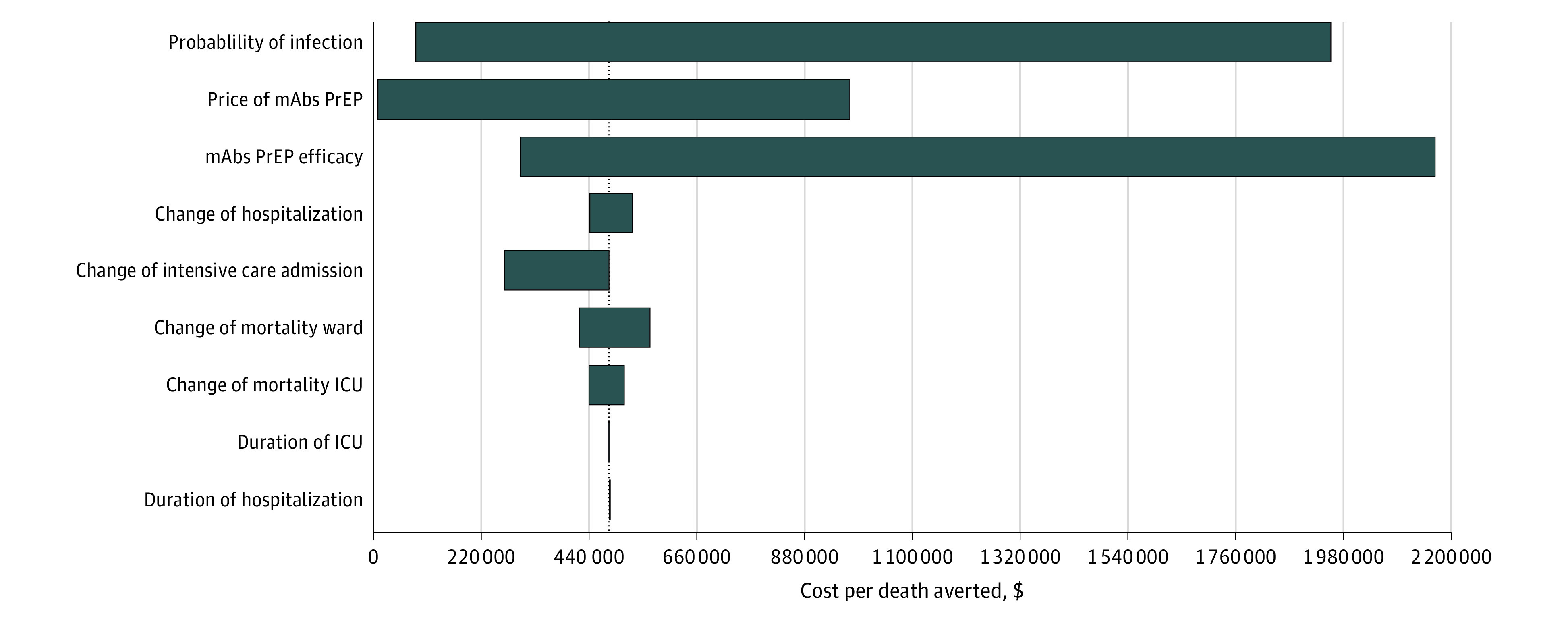
One-way Sensitivity Analysis of the Cost-effectiveness Ratio Per Quality-Adjusted Life Year We calculated the cost-effectiveness ratio per quality-adjusted life-year gained of no monoclonal antibodies pre-exposure prophylaxis (mAbs PrEP) vs 50% mAbs PrEP effectiveness, high mAbs PrEP price, and low SARS-CoV-2 infection probability as baseline comparator. The following key input parameters were independently varied: infection probability (0.01-0.2), effectiveness (10%-100%), drug price ($100-$5,000), admission probability (ward and ICU), mortality, and length of hospital stay (±50%). A medium number of quality-adjusted life-years (3.7) was used. The dotted line indicates the cost-effectiveness ratio per quality-adjusted life-year. The bars depict the variation in the cost-effectiveness ratio when parameters are independently varied. ICU indicates intensive care unit.

Our 1-way sensitivity analysis pointed out 9 key parameters of our model. Our results showed that the cost-effectiveness ratios are most affected by the SARS-CoV-2 infection probability, the mAbs PrEP drug price, and mAbs PrEP effectiveness (Figure 3). With a 50% effectiveness of mAbs PrEP and low SARS-CoV-2 infection probability, a mAbs PrEP price of $180 or less can result in cost-effectiveness ratios lower than $88 000 per QALY. However, a high (0.2) probability of infection or 100% effectiveness with a $2750 PrEP price does not result in scenarios with ratios lower than $88 000. The other parameters influenced our model outcome to a lesser extent when varied ±50%.

## Discussion

Our decision analytic model showed that within the context of a high SARS-CoV-2 probability, mAbs PrEP provision was cost-saving when provided to individuals at increased risk of severe COVID-19 if drug prices decrease to $275 and effectiveness is 75% or higher. Cost-effective scenarios (<$22 000 per QALY gained) can still be reached for all individuals when mAbs PrEP effectiveness is at 75%, and drug prices are $550. The data suggest that with a lower PrEP effectiveness, drug prices should be revised downwards. In the Netherlands, if higher cost-effectiveness ratios of $44 000 to $88 000 per QALY gained are accepted, translating the possibility of higher drug prices could be acceptable, depending on the underlying effectiveness.^21^

At the beginning or peak of an epidemic wave, higher infection probabilities can be expected in which mAbs PrEP provision had the most substantial estimated reducing effect on new SARS-CoV-2 infections, admissions, deaths, and health care utilization costs. Similar findings were found within a mAbs postexposure prophylaxis decision analytic modeling study where a larger transmission risk resulted in a greater reduction in COVID-19-related costs.^22^ Even if the effectiveness would lower over time, our model still demonstrated a substantial health gain when implemented. Drug prices should therefore be further negotiated with the advocacy of governments and patient organizations (presently estimated at $2750 in the Netherlands). Strong advocacy in lowering drug prices resulted in a reduction in the hepatitis C direct-acting antivirals price several years ago, which can be achieved with similar support for other drugs.^23,24^

Our data are an underrepresentation of the overall effect of implementing mAbs PrEP as we do not model SARS-CoV-2 transmission or a shorter disease duration. MAbs PrEP, however, will have an expected preventative effect on transmission and thereby reduce the number of new infections. Moreover, hospital admissions with possible additional antiviral therapies are not required and therefore further lower costs. Additionally, overall disease duration will be shorter resulting in less work absence and productivity loss. As our analysis is performed from a third-party payer’s perspective, costs of absence of work, productivity loss, and direct patient cost have not been taken into account. However, further inclusion of individuals’ perspectives would only increase the mAbs PrEP cost-effectiveness.

Presently, numerous countries, including Canada, France, Israel, and the US, have rolled out mAbs PrEP programs with tixagevimab-cilgavimab as a management strategy to prevent COVID-19 in high-risk populations.^12,14,25^ The forthcoming clinical data from these countries, representing sublineages of the Omicron variant, are highly encouraging in mAbs PrEP use and show substantial reductions in infections, hospital admissions, and deaths.^12,14,25,26,27^ Other countries, such as the United Kingdom and the Netherlands, are still hesitant to roll out mAbs PrEP, citing a lack of effectiveness data on currently circulating variants.^7,8^ The SARS-CoV-2 viral landscape will inevitably change, and should not hamper using lifesaving drugs as they can be moderately effective. Therefore, our study provides a state-of-play adding to the emerging clinical data aiming to show policy makers that, notwithstanding a constant varying viral landscape, mAbs as COVID-19 PrEP does provide benefits and should be implemented.^12,14,25^

The emergence of resistance-associated variants lowering mAbs PrEP effectiveness is another relevant barrier in mAbs PrEP implementation. Our model showed that a lower effectiveness (eg, below 50% with moderate QALYs gained per death averted) resulted in higher (>$88 000) cost-effectiveness ratios. Especially, when infection probabilities are also low, cost-effectiveness ratios increase substantially. We and others have shown the emergence of resistance-associated variants during treatment of COVID-19 with mAbs.^16,28^ However, there is not yet an understanding of any potential viral fitness costs of these variants, and transmission of these variants has not yet been observed at scale. Nonetheless, caution is required, and studies should aim for phylogenetic analysis to see whether emerging resistant variants resulting from mAbs PrEP are fit enough for transmission. Moreover, newer mAbs should be targeted to more highly conserved spike protein regions and given in combination therapy similar to HIV and hepatitis C.

### Strengths and Limitations

Our study has several strengths. First, to our knowledge, this is the first analysis to estimate the cost-effectiveness of mAbs PrEP use among high-risk individuals and can provide a valuable tool for deciding on SARS-CoV-2 PrEP implementation. Second, we assessed data from a large clinical cohort (TURN-COVID) of high-risk individuals to parameterize our model.^16^ Third, to estimate health care costs, we used the combination of self-completed health care utilization questionnaires and EHR data of this high-risk patient population. Lastly, the SARS-CoV-2 viral landscape is constantly changing resulting in a varying mAbs PrEP effectiveness resulting in lower or higher hospitalization and mortality rates. Our study, therefore, accounted for this variability by performing a sensitivity analysis in which we varied these parameters.

Our study has several limitations. First, our study may underestimate the cost of patients with COVID-19 who died within the 90-day period due to missing self-completed data collection. Given that the highest cost drivers were inpatient days, and these data were available from EHRs, our results are likely to be minimally impacted by the lack of this self-completed data. Moreover, most individuals died within the first 2 weeks after hospitalizations thus, no additional health care costs were used after that. Second, our study population was heterogeneous; therefore, the number of QALYs gained per death averted was difficult to estimate. To address this, we provided a range in the number of QALYs gained assigned to each death averted, which can be extrapolated to other populations. Third, due to self-completed questionnaires regarding 90 days of health care utilization, there could be a recall bias. However, our highest cost components, inpatient days, are drawn from the EHRs. Fourth, our analysis only included the short-term costs after COVID-19. However, the addition of long-term costs (for example, 180 days) would likely increase the cost-effectiveness of mAbs PrEP, particularly as some individuals require a longer rehabilitation period after COVID-19.^29^ Additionally, our health care utilization and costs are focused on primary SARS-CoV-2 infections and do not take reinfections into account. Because reinfections increase health care utilization and consequently costs, mAbs PrEP would be more cost-effective during an annual seasonal peak.

## Conclusions

In conclusion, mAbs PrEP can be cost-saving or cost-effective depending on the effectiveness, cost, and probability of infection. Given that at the beginning of each epidemic wave, an understanding of the effectiveness and infection probability is unknown, one should advocate for drug prices ensuring cost-savings or cost-effectiveness of mAbs PrEP provision moving forward.

## Notes

Supplement 1. **eFigure 1. **Schematic Representation of Decision Analytic Model **eAppendix. **Data Collection and Cost Calculation **eTable 1. **Input Parameters Decision Analytic Model and Health Care Resources **eTable 2.** Short-term (90 Days) Health Care Utilization Costs Derived From 636 High-risk Patients of the TURN-COVID Cohort **eFigure 2. **Short-term Cost-effectiveness Ratios Per Quality-Adjusted Life Year (QALY) Gained
eReferences

Supplement 2.
Nonauthor Collaborators

Supplement 3.
Data Sharing Statement
