# Supplementary material for: Health Outcomes and Cost-effectiveness of Monoclonal SARS-CoV-2 Antibodies as Pre-exposure Prophylaxis
Source: JAMA Netw Open. 2023 Jul 6;6(7):e2321985. doi: 10.1001/jamanetworkopen.2023.21985 (PMC10326646; doi:10.1001/jamanetworkopen.2023.21985)
Supplement: Supplement 1. — eFigure 1. Schematic Representation of Decision Analytic Model eAppendix. Data Collection and Cost Calculation eTable 1. Input Parameters Decision Analytic Model and Health Care Resources eTable 2. Short-term (90 Days) Health Care Utilization Costs Derived From 636 High-risk Patients of the TURN-COVID Cohort eFigure 2. Short-term Cost-effectiveness Ratios Per Quality-Adjusted Life Year (QALY) Gained eReferences [file jamanetwopen-e2321985-s001.pdf]

Supplemental Online Content

Popping S, Nichols BE, Appelman B, et al; TURN-COVID study group. Health outcomes and costs-effectiveness of monoclonal SARS-CoV-2 Antibodies as pre-exposure prophylaxis. *JAMA Netw Open*. 2023;6(7):e2321985. doi:10.1001/jamanetworkopen.2023.21985

eFigure 1. Schematic Representation of Decision Analytic Model
eAppendix. Data Collection and Cost Calculation
eTable 1. Input Parameters Decision Analytic Model and Health Care Resources
eTable 2. Short-term (90 Days) Health Care Utilization Costs Derived From 636 High-risk Patients of the TURN-COVID Cohort
eFigure 2. Short-term Cost-effectiveness Ratios Per Quality-Adjusted Life Year (QALY) Gained
eReferences

This supplemental material has been provided by the authors to give readers additional information about their work.

© 2023 Popping S et al. *JAMA Network Open.*

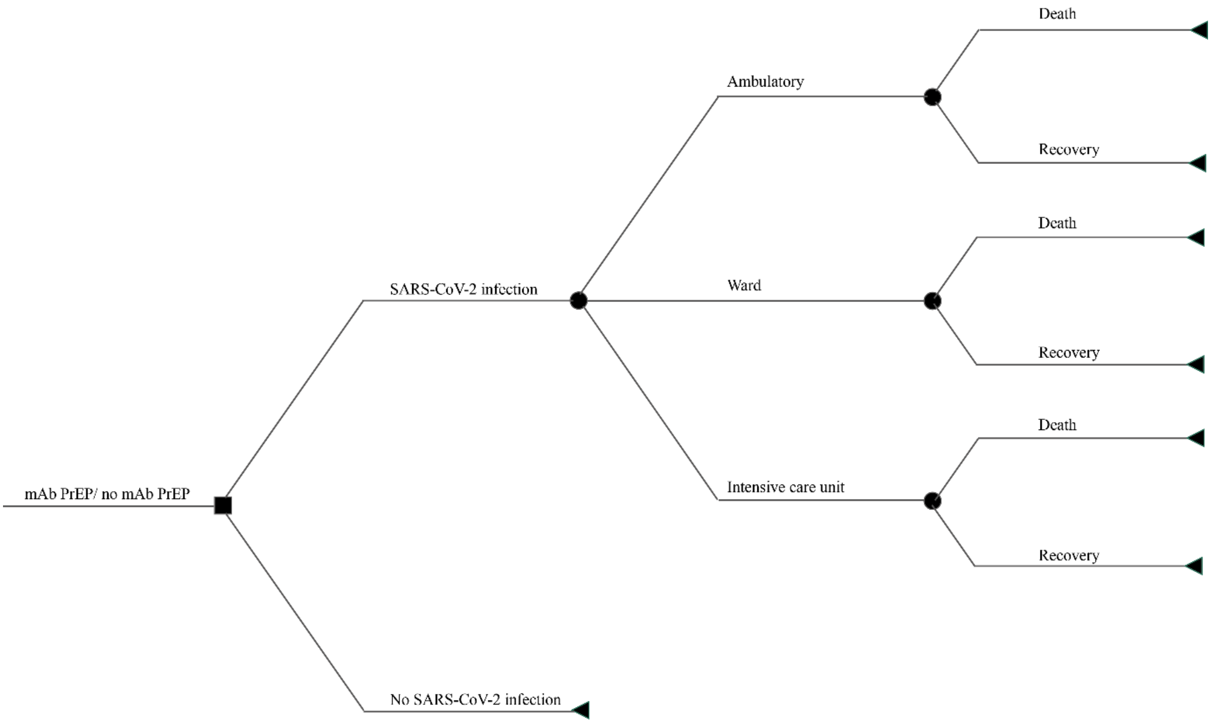

eFigure 1. Schematic Representation of Decision Analytic Model
The square represents a decision node of providing mAbs PrEP followed by a series of chance nodes (circles) and eventually terminal nodes (triangles).

© 2023 Popping S et al. *JAMA Network Open.*

eAppendix. Data Collection and Cost Calculation
Data collection

Clinical and healthcare utilization data was collected from self-reported questionnaires and Electronical Health Records (EHRs). A self-completed questionnaire was requested on day 90, capturing healthcare utilization during the 90-day period following COVID-19. Additionally, the 90-day period preceding COVID-19 were collected on day 28.

Self-reported questionnaires were sent out via email, and participants were reminded via a telephone call by our data collection team to fill-out the questionnaires. Our self-reported questionnaires included questions on inpatient days (ward, intensive care unit (ICU), and rehabilitation), Emergency department visits, paramedical care, care facilitated by the general practitioners' offices, and co-medication use (see also eTable1). In addition, data was subtracted from the EHRs, including inpatient days (ward, intensive care unit (ICU), and rehabilitation), Emergency department visits, and co-medication use.

EHR data collection was in collaboration with data collectors from the "Stichting HIV Monitoring" (SHM).[11,12] The SHM has extensive experience collecting clinical data of people living with HIV from EHRs for many years. Their HIV work consists of collecting all clinical and therapeutical data from 98% of people living with HIV in care in the Netherlands. They have developed a data manual from data collection, which could be further updated for the TURN-COVID study. We stored all collected data in DataCaptree (LogicNets, Amsterdam, The Netherlands). We compared and complemented self-reported and EHR data for our final data set.

Cost calculation

We have built a model to calculate the healthcare utilization costs per individual of our clinical cohort (TURN-COVID). In the model, all healthcare utilization data from our final data set was multiplied by the healthcare resource unit prices over a 90-day period. Healthcare resource unit prices were mostly based on recommended prices by the Dutch Healthcare institute (Nederlandse Zorg Instituut) and the Dutch Healthcare Authority (Nederlandse Zorg Autoriteit, NZa).[7] We used unit prices from the year 2022 and recommended unit prices from before the year 2022 were indexed based on annual Dutch inflation as stated by the Central Bureau for Statistics (CBS; statistics Netherlands).[8] If unit prices were not recommended by the Dutch Healthcare institute or the Dutch Healthcare Authority (e.g., uninsured care), we used the average of four different care providers.

As all unit prices were based on Dutch prices, we converted euros to dollars using a 1.1 conversion rate (May 8th 2023). To account for COVID-19-related costs we compared the healthcare utilization costs in the 90-days prior COVID-19 with the 90-day period after COVID-19. The total mean costs were calculated per patient category and outcome

© 2023 Popping S et al. *JAMA Network Open.*

**Input parameters decision analytic model**		
Transition probabilities		
SARS-CoV-2 infection	0.04 - 0.18	¥1
Ambulatory (patient with mild COVID-19)	0.81	¥
Ward (patient with moderate COVID-19)	0.14	¥,2
Intensive care unit (patient with severe/critical COVID-19)	0.05	¥,3,4
Mortality ambulatory patient	0.05	¥
Mortality ward patient	0.10 - 0.14	¶,4
Mortality intensive care unit patient	0.08 - 0.21	¶,4,5
Price per unit ($)		
SARS-CoV-2 rapid test	1.98	1
SARS-CoV-2 PCR	77	1
Single dose of mAbs PrEP	275 – 2,750	¥
Monoclonal antibody PrEP at 100% effectiveness		
Reduction probability of SARS-CoV-2 infection	70%	1,2,6
Reduction in ward admission	85%	1,2
Reduction in intensive care unit admission	94%	1,2,6
Reduction in overall mortality	65%	2,6
**Healthcare resources**		
Price per unit ($)		References
Hospital admission, ward day	603	7,8
Hospital admission, intensive care unit day	2,552	7,8
Emergency department visit	318	7,8
Emergency department visit, including ambulance transport	652	7,8
Out-patient appointment, several specialists	100 - 167	7,8
Medical rehabilitation, outpatient appointment	194	7,8
Medical rehabilitation, inpatient day	583	7,8
Nursing home, inpatient day	212	7,8
First-line care by the general practitioners' office		
Single, double, home visits	6 – 30	8,9
Consultation by phone	6	8,9
Palliative visits	91	8,9
Paramedical visit	31 – 50	1
Psychologist	94 - 112	1
Speech therapy visit	37 - 78	1
Social worker appointment	72	1
Alternative medicine appointment	44 – 121	1
Co-medication, per day	0.03 – 82	10

eTable 1. Input Parameters Decision Analytic Model and Health Care Resources. All unit prices are from the year 2022 and mostly based on recommended prices by the Dutch Healthcare institute (Nederlandse Zorg Instituut) and the Dutch Healthcare Authority (Nederlandse Zorg Autoriteit, NZa).[7] Recommended unit prices prior to the year 2022 were indexed based on annual Dutch inflation as stated by the Central Bureau for Statistics (CBS; statistics Netherlands).[8] Prices were converted from euro to dollar using a 1.1 conversion rate (8th of May 2023). [1] Average based on 4 different providers, ¥ Estimate, ¶ TURN-COVID data.

© 2023 Popping S et al. *JAMA Network Open.*

	Ambulatory (155)	Ward (366)	Intensive care unit (115)
**Short term healthcare utilisation costs**			
Recovered, median [IQR]	$0 [0 - 138]	$4,498 [$2,482 - $7,836]	$27,309 [$15,857 – $45,630]
Recovered, mean	$166	$6,742	$39,313
Recovered, range	-$1,101 - $2,374[1]	$603 – $56,060	$3017 - $197,189
Death, median [IQR]	-	$4,220 [$2,411 - $6,631]	$34,984 [$21,551 – $50,633]
Death, mean	$ 275[2]	$5,202	$36,784
Death, range	-	$603 - $16,878	$31,55- $79,112

eTable 2. Short-term (90 Days) Health Care Utilization Costs Derived From 636 High-risk Patients of the TURN-COVID Cohort

[1] Among few individuals (n=9) the healthcare utilisation costs were lower in the 90-day period following SARS-CoV-2 infection compared to the period preceding infection

[2] No healthcare utilisation data available, therefore we based these costs on the mean costs of ambulatory recovered patients with an additional palliative visit and two consultations by the general practitioner

© 2023 Popping S et al. *JAMA Network Open.*

Low number of QALY gained per death averted 1.865

Moderate number of QALY gained per death averted 3.73

High number of QALY gained per death averted 7.46

Legend: € <20 000 | € 20 000 - 80 000 | € > 80 000

Low SARS-CoV-2 probability

Low number of QALY gained per death averted 1.865

PrEP Price €				
2500	1614666	867751	637147	539932
2000	1289463	690606	505364	427138
1000	639057	336315	241799	201549
500	313854	159170	110016	88754
250	151253	70597	44125	32357
PrEP Efficacy	25%	50%	75%	100%

Moderate number of QALY gained per death averted 3.73

PrEP Price €				
2500	807333	433875	318573	269966
2000	644731	345303	252682	213569
1000	319529	168158	120899	100774
500	156927	79585	55008	44377
250	75626	35299	22063	16178
PrEP Efficacy	25%	50%	75%	100%

High number of QALY gained per death averted 7.46

PrEP Price €				
2500	403666	216938	159287	134983
2000	322366	172651	126341	106784
1000	159764	84079	60450	50387
500	78464	39793	27504	22188
250	37813	17649	11031	8089
PrEP Efficacy	25%	50%	75%	100%

High SARS-CoV-2 probability

Low number of QALY gained per death averted 1.865

PrEP Price €				
2500	349988	178853	124659	101287
2000	277721	139487	95374	76221
1000	133186	60756	36804	26090
500	60919	21390	7519	1025
250	24785	1708	CS	CS
PrEP Efficacy	25%	50%	75%	100%

Moderate number of QALY gained per death averted 3.73

PrEP Price €				
2500	174994	89426	62329	50643
2000	138860	69744	47687	38111
1000	66593	30378	18402	13045
500	30459	10695	3759	512
250	12393	854	CS	CS
PrEP Efficacy	25%	50%	75%	100%

High number of QALY gained per death averted 7.46

PrEP Price €				
2500	87497	44713	31165	25322
2000	69430	34872	23843	19055
1000	33297	15189	9201	6523
500	15230	5348	1880	256
250	6196	427	CS	CS
PrEP Efficacy	25%	50%	75%	100%

eFigure 2. Short-term Cost-effectiveness Ratios Per Quality-Adjusted Life Year (QALY) Gained

The panels in the figure shows a different number of QALYs gained per death averted representing different patient populations with underlying diseases and their life-expectancy.[13] Abbreviations: CS – cost-saving ($<0).

© 2023 Popping S et al. *JAMA Network Open.*

eReferences

1. Kertes J, Shapiro Ben David S, Engel-Zohar N, et al. Association Between AZD7442 (Tixagevimab-Cilgavimab) Administration and Severe Acute Respiratory Syndrome Coronavirus 2 (SARS-CoV-2) Infection, Hospitalization, and Mortality. *Clin Infect Dis.* 2022. doi: 10.1093/cid/ciac625
2. Kaminski H, Gigan M, Vermorel A, et al. COVID-19 morbidity decreases with tixagevimab-cilgavimab preexposure prophylaxis in kidney transplant recipient nonresponders or low-vaccine responders. *Kidney Int.* 2022;102(4):936-938. doi: 10.1016/j.kint.2022.07.008
3. Wiersinga WJ, Rhodes A, Cheng AC, Peacock SJ, Prescott HC. Pathophysiology, Transmission, Diagnosis, and Treatment of Coronavirus Disease 2019 (COVID-19): A Review. *JAMA.* 2020;324(8):782-793. doi: 10.1001/jama.2020.12839
4. Stichting-nice.nl. *COVID-19 in Dutch Intensive Care Units; Patient characteristics and outcomes compared with pneumonia patients in the ICU from 2017-2019.* 2022.
5. Dongelmans DA, Termorshuizen F, Brinkman S, et al. Characteristics and outcome of COVID-19 patients admitted to the ICU: a nationwide cohort study on the comparison between the first and the consecutive upsurges of the second wave of the COVID-19 pandemic in the Netherlands. *Ann Intensive Care.* 2022;12(1):5. doi: 10.1186/s13613-021-00978-3
6. Levin MJ, Ustianowski A, De Wit S, et al. Intramuscular AZD7442 (Tixagevimab-Cilgavimab) for Prevention of Covid-19. *N Engl J Med.* 2022;386(23):2188-2200. doi: 10.1056/NEJMoa2116620
7. Zwaap J KS, van der Meijden C, Staal P, van der Heiden L. Cost-effectiveness in practice; Kosteneffectiviteite in de praktijk. https://www.zorginstituutnederland.nl/publicaties/rapport/2015/06/26/kosteneffectiviteit-in-de-praktijk. Published 2015. Accessed.
8. Statistics Netherlands CBvds. Inflation numbers (Inflatie cijfers). https://opendata.cbs.nl/#/CBS/nl/. Published 2022. Accessed.
9. Dutch healthcare authority (Nederlandse Zorgautoriteit N. Performance and tariff decision for general practitioner care and multidisciplinary care (Prestatie - en tariefbeschikking huisartsenzorg en multidiscipliaire zorg) 2022 - RB/REG-22622-04. Nederlandse Zorgautoriteit https://puc.overheid.nl/nza/doc/PUC_694745_22/1/. Published 2022. Accessed.
10. National Healthcare Institute ZN. Medication cost (Medicatie Kosten). www.medicijnkosten.nl. Published 2022. Accessed2022.
11. Smit C, Boyd A, Rijnders BJA, et al. HCV micro-elimination in individuals with HIV in the Netherlands 4 years after universal access to direct-acting antivirals: a retrospective cohort study. *Lancet HIV.* 2021;8(2):e96-e105. doi: 10.1016/s2352-3018(20)30301-5
12. Wymant C, Bezemer D, Blanquart F, et al. A highly virulent variant of HIV-1 circulating in the Netherlands. *Science.* 2022;375(6580):540-545. doi: 10.1126/science.abk1688
13. Wouterse B, Ram F, van Baal P. Quality-Adjusted Life-Years Lost Due to COVID-19 Mortality: Methods and Application for The Netherlands. *Value Health.* 2022;25(5):731-735. doi: 10.1016/j.jval.2021.12.008

© 2023 Popping S et al. *JAMA Network Open.*
