# Supplementary material for: Health Outcomes and Cost-effectiveness of Monoclonal SARS-CoV-2 Antibodies as Pre-exposure Prophylaxis
Source: JAMA Netw Open. 2023 Jul 6;6(7):e2321985. doi: 10.1001/jamanetworkopen.2023.21985 (PMC10326646; doi:10.1001/jamanetworkopen.2023.21985)
Supplement: Supplement 3. — Data Sharing Statement [file jamanetwopen-e2321985-s003.pdf]

Data Sharing Statement

Popping. Health Outcomes and Cost-effectiveness of Monoclonal SARS-CoV-2 Antibodies as Pre-exposure Prophylaxis. *JAMA Netw Open*. Published July 06, 2023.
doi:10.1001/jamanetworkopen.2023.21985

Data
Data available: No

Additional Information
Explanation for why data not available: All data is available in the manuscript and supplement. The original data set is, however, unavailable.
